# COVID-19 Pandemic in India: Through the Lens of Modeling

**DOI:** 10.9745/GHSP-D-21-00233

**Published:** 2021-06-30

**Authors:** Giridhara R. Babu, Debashree Ray, Ritwik Bhaduri, Aritra Halder, Ritoban Kundu, Gautam I. Menon, Bhramar Mukherjee

**Affiliations:** a Indian Institute of Public Health, Public Health Foundation of India, Bengaluru, India.; b Johns Hopkins Bloomberg School of Public Health, Baltimore, MD, USA.; c Indian Statistical Institute, Kolkata, India.; d Social and Decision Analytics Division, Biocomplexity Institute, University of Virginia, USA.; e Ashoka University, Sonepat, India.; f Institute of Mathematical Sciences, Chennai, India.; g University of Michigan School of Public Health, Ann Arbor, MI, USA.

## Abstract

We reflect on and review India's COVID-19 pandemic response through the lens of modeling and data. The lessons learned from the Indian context may be beneficial for other countries.

## Key Messages

India has devised innovative strategies to reduce the spread of COVID-19 within the constraints of a low-resource setting. India has also made some questionable policy decisions. Lessons learned from the Indian experience for public health, health care, and data infrastructure can be globally valuable. In this commentary, as a team of public health data scientists engaged in modeling the pandemic since early 2020, we reflect on India's journey over the past 1 year.

## INTRODUCTION

India, the world's largest democracy, declared its first confirmed case of severe acute respiratory syndrome coronavirus-2 (SARS-CoV-2) infection on January 30, 2020. It currently reports Asia's largest number of coronavirus disease (COVID-19) infections and deaths (27.7 million reported cases and 322,384 reported deaths as of May 28, 2021).^1^ The actual numbers for both infections and deaths likely far exceed what are officially reported.

In the past year, India has devised some innovative strategies aiming to reduce COVID-19 spread within the constraints of a low-resource setting. It has also made some questionable policy decisions. Lessons learned from the Indian experience for public health, health care, and data infrastructure can be globally valuable. In this commentary, as a team of public health data scientists engaged in modeling the pandemic since early 2020, we reflect on India's journey over the past year.

## THE LANDSCAPE OF EPIDEMIOLOGICAL MODELS

Epidemiological models help public health planners gauge the future predicted trajectory of epidemics, providing forecasts or estimates for the daily number of infections, hospitalizations, and deaths. Models operate under various assumptions. They can incorporate hypothetical intervention scenarios and assess their relative impact on disease transmission. Because they may help us calibrate our expectations and resource needs for the future, predictive models have drawn significant attention from the media and the public.^2^

### Types of Commonly Used Models

There have been many models proposed for the spread of COVID-19 in India. These models can be broadly categorized into 2 genres: exponential/Poisson-type regression models and compartmental epidemiological models. For instance, Ranjan^3^ and Gupta and Shankar^4^ use the classical exponential model on the daily case counts. The compartmental models include variations of the susceptible-infected-removed (SIR) model. Such models are guided by a set of differential equations relating to the number of susceptible people, the number of infected people (cases), and the number of people who have been removed (either recovered or dead) at any given time. One extension of the SIR model is the susceptible-exposed-infected-removed (SEIR) model that incorporates an additional compartment of truly exposed people which is latent or unobserved. Ray et al.^5^ provide a summary of these models and their basic assumptions; Sarkar et al.^6^ provide an early comprehensive review, and Purkayastha et al.^7^ provide a head-to-head comparison of 5 different models for forecasting, with a focus on India. Such models differ in terms of the data they use, ranging from simple case counts to age-sex demography, age-specific contact networks, and mobility data. Some, but not all, models are transparent, explicitly stating assumptions, making their code available, and updating their predictions regularly.^5^^,^^8^ Such constant recalibration and updating are critical, given that the reality on the ground changes rapidly.

Providing uncertainty estimates for point predictions is also essential since the predictive ability of these models deteriorates rapidly over times longer than a few weeks. Several authors have pointed out that India is heterogeneous. Allowing different state- and district-level model predictions from multiple groups to aggregate toward national-level predictions is a better approach.^9^ It would be audacious to claim the superiority of a single model or base public health decisions solely on one.^10^ In principle, ensemble methods that average over predictions across multiple models should provide predictions that benefit from aggregated learning.^11^ However, for dynamic systems used for modeling the virus transmission, aggregating results from models with diverse assumptions, structure, and inputs may lead to a lack of interpretability.

Allowing different state- and district-level model predictions from multiple groups to aggregate toward national-level predictions is a better approach to predicting virus transmission.

### Mismeasured Case and Death Counts

While projections based on reported daily case-counts have received the most attention, the differential availability of testing and the high rates of false negatives in the rapid antigen tests (30%–40%) and RT-PCR tests (15%–30%) mandate that investigators either explicitly account for selective and imperfect testing or conduct careful sensitivity analysis.^12^^,^^13^ Serosurveys and epidemiological models have confirmed a high degree of covert infections for India, and a reasonable estimate would suggest that more than 90% of infections remain unreported.^12^ A recent preprint^12^ shows that the estimated case underreporting factor for India using data from April 1 to August 31, 2020, is approximately between 10 and 20, with the death underreporting factor estimated at approximately between 2 and 5 as of September 1, 2020. These estimates are obtained from an extension of the SEIR model accounting for the high false-negative rates of diagnostic tests (misclassification bias) and the symptom-based administration of these tests (selection bias).^12^ The infection fatality rate (IFR) for India is estimated to be around 0.1% (using officially reported death counts), whereas the reported case fatality rate (CFR) is 1.4% at the end of the year 2020. There is substantial heterogeneity in the case counts across Indian states. The Figure exhibits heat maps on a logarithmic scale, indicating the number of confirmed cases on July 1, September 1, and November 1, 2020, during the first pandemic wave. These demonstrate the very inhomogeneous spread of COVID-19 in India, which centered largely around the major urban agglomerations in a small number of states over much of the early and intermediate period, expanding only later across the country.

**FIGURE:**
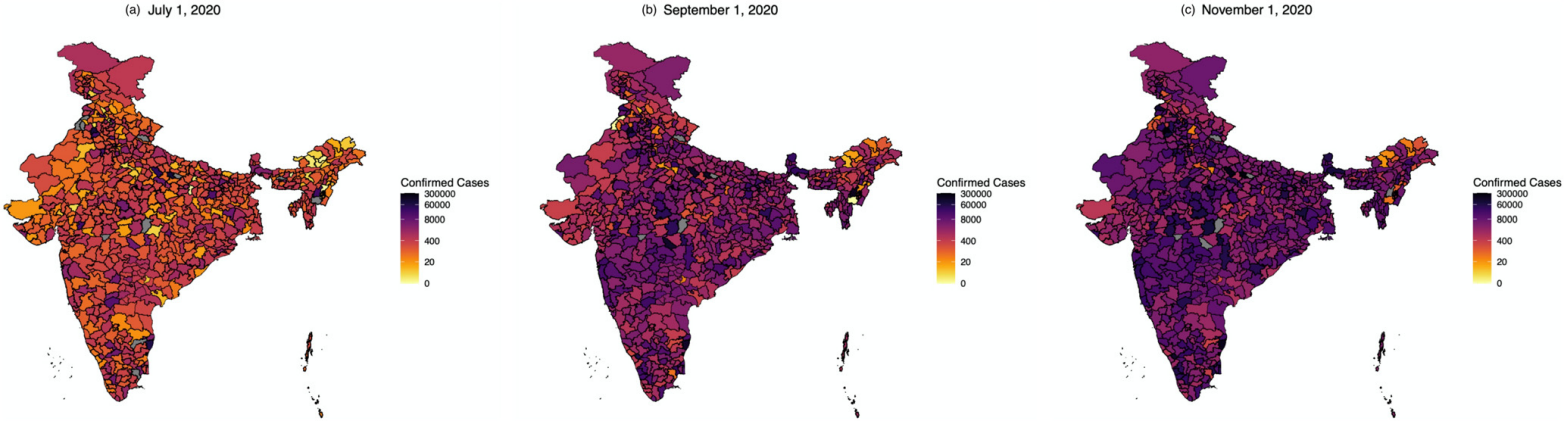
Case Counts in India Using District-Level Data^a^ as Reported on (a) July 1, (b) September 1, and (c) November 1, 2020, Shown as a Heat Map in Logarithmic Scale ^a^ From covid19india.org.

### Expanded Models Using Additional Information on Vaccines and Variants

Newer models leveraging information from serosurveys have appeared (e.g., the well-known Institute for Health Metrics and Evaluation model in the United States).^14^ In the Indian context, Mandal et al.^15^ use a compartmental model to project the demand for hospitalization, accounting for population immunity estimated by serosurveys. With emerging new variants of SARS-CoV-2 (e.g., B.1.1.7 from the United Kingdom [UK], B.1.351 from South Africa, or P.1 from Brazil), many countries are adopting models that additionally incorporate differential transmissibility and time-varying reproduction numbers of these variants (Table).^16^^–^^22^ Now that community-wide vaccination programs are underway in many countries, some of these models also consider different vaccine effectiveness profiles.^16^^–^^18^ One straightforward approach is to use the traditional SIR or SEIR model, where each compartment is stratified by vaccine status and infecting variant, and to consider time-varying vaccination rates and variant transmission rates.^17^ To our knowledge, the impacts of both vaccines and new virus variants have not yet been incorporated in any model from India.

**TABLE.** COVID-19 Epidemiological Models Incorporating Effects of Different Variants of SARS-CoV-2 and/or Impact of Vaccines

Model Type	Location of Data Used	Research Question	Key Variables/Parameters Considered	Reference
Two-variant compartmental model	USA	Assess the effect of VOC B.1.1.7 on the US pandemic trajectory in Jan-Apr 2021	SARS-CoV-2 immunity from previous infection; time-varying reproduction numbers of current variants and VOC; potential impact of community vaccination	Galloway et al.^16^
SIR model with compartments stratified by age, vaccine status, and infecting strain	Washington, USA	Project the timing and intensity of subsequent waves of infection	Time-varying, age-stratified effects of non-pharmaceutical interventions; different case thresholds for triggering and relaxing partial lockdowns; different vaccination rates and vaccine efficacy profiles; different infectivity rates of current variant and VOC B.1.1.7	Reeves et al.^17^
An extended θ-SIR model	Italy	Assess the impact of VOC B.1.1.7 and vaccination campaigns on spread of COVID-19	Different numbers of SARS-CoV-2 variants and vaccines; time-varying reproduction numbers; differential immunity depending on dose of vaccine	Ramos et al.^18^
An extended SEIR model(“UVA PatchSim model”)	Virginia, USA	Assess the preliminary effect of vaccines and potential effect of new variants on the trajectory of cases	Seasonal effects, including effect from pandemic fatigue; impact of VOC B.1.1.7; impact of community vaccination	University of Virginia Department of Health^19^
An extended SEIR model (“Behavioral SEIR”)	USA; UK	Forecast the evolution of the epidemic	Seasonal variation in transmission rate; pandemic fatigue; spread of the new variant (same seasonal pattern but different transmissibility)	Atkeson^20^
An extended SEIR model	Brazil	Forecast disease transmission behaviour under 2 SARS-CoV-2 dominant strains	Time-varying reproduction numbers of the original SARS-Cov-2 D614 and its dominant G614 variant; different incubation rates	Celaschi^21^
Renewal equation based semi-mechanistic model	England	Compare reproduction numbers of VOC with non-VOC	Time-varying reproduction numbers of current variants and VOC B.1.1.7; hotspots of infection; true positive rate adjusted SGTF frequencies (proxy for VOC frequencies)	Volz et al.^22^
Age-structured SEIR model	India	Assess optimal vaccine allocation strategies	Different age-stratified vaccination strategies and rates; different efficacies and types of immunity from vaccines	Foy et al.^25^

Abbreviations: θ, instantaneous ratio of new detected infectious cases per unit time over the total; COVID-19, coronavirus disease; SARS-CoV-2, severe acute respiratory syndrome coronavirus-2; SEIR, susceptible-exposed-infected-recovered; SGTF, S-gene target failures; SIR, susceptible-infected-recovered; VOC, variant of concern.

With emerging new virus variants, many countries are adopting models that incorporate differential transmissibility and time-varying reproduction numbers of these variants.

### Resource Allocation Models

When compared to prediction models for case-/death-/hospitalization-counts, less public attention has been given to resource allocation models used to deploy health care resources like estimating oxygen supply or the number of hospital beds^23^ or risk stratification using individual-level data.^24^ Modeling approaches are critical as India rolls out its nationwide vaccine distribution plan based on hierarchical risk prioritization. Such models based on ecological-level data have been recently proposed.^25^

## MYSTERIES UNEXPLAINED BY MODELS

### Plausible Causes of Lower Case-Fatality Rates in India

The comparatively low numbers for COVID-19 fatalities per million population in India provide some cheer. As of March 30, 2021, India had 119 deaths per million, in comparison to numbers for Brazil (1,507 deaths per million), Germany (926 deaths per million), the UK (1,892 deaths per million), and the United States (1,585 deaths per million).^1^ However, India has higher reported fatalities per million compared to neighboring countries: 65 (Afghanistan), 59 (Myanmar), 106 (Nepal), and 66 (Pakistan).^1^ Some of this can be attributed to India's relatively young population. India has a median age of 29 years while Bangladesh and Pakistan have median ages of 27 and 23 years, respectively.^26^ These numbers should be compared to the median ages of 47 years in Germany and 38 years in the United States. The proportion of the population aged 65 and older who are most susceptible to COVID-19 severity is 6.4% in India, 5.2% in Bangladesh, 4.3% in Pakistan. The corresponding numbers are 21.6% in Germany and 16.5% in the United States.^26^

There have been suggestions that South Asian populations may be protected from more severe forms of the disease for various reasons. These theories include the possibility that infections from other types of coronaviruses in early life, leading to a stronger innate immune response.^27^ The South Asian microbiome may differ in qualitative ways from the Western ones,^28^ compulsory childhood vaccination programs may play a role,^29^ and a genetic component to protect from the disease may exist.^30^ The large number of patients detected by contact tracing who are asymptomatic at the time of testing suggests an overall milder impact of the disease.^31^ Plausible explanations in support of India's low fatality rates include cross-immunity, genetics, prior vaccination, younger population, a predominantly outdoor lifestyle in rural areas, and plenty of outside air circulating through homes in urban settings. India is nearly 70% rural whereas European countries are overwhelmingly urban, facilitating the spread of the virus. However, all such hypotheses are conjectural at this point, and no causal association has been established.

Conjectural theories explaining causes of India's comparatively low fatality rates include cross-immunity, genetics, prior vaccination, younger population, and a predominantly outdoor lifestyle in rural areas.

Set against these hypotheses is the possibility that any innate advantage to the South Asian population is illusory, arising from inadequate counting of COVID-19 deaths.^32^ There is certainly evidence that many deaths due to COVID-19 have not been classified as COVID-19 deaths (e.g., attributing patients' underlying conditions or comorbidities as the cause of death).^33^ Evidence of deaths with symptoms suggestive of COVID-19 infection comes from on-the-ground reporting from crematoria and burial grounds,^34^ detailed citizen-science-driven studies of obituaries,^35^ evidence from the patients' families, and deathcertificates.^35^ Estimates of COVID-19 death undercounting range from a factor of 1.5 to 5.^36^ A holistic measure of excess mortality due to the pandemic could have been obtained by estimating the excess over all-cause mortality in non-COVID years if comprehensive historical death data were available.^37^ There is every reason to believe that in India, as is the case elsewhere, there have been excess deaths indirectly caused by the pandemic, due, for example, to delays in reaching care or compromised capacity for hospital care. On the other hand, confounders such as the abrupt national lockdown in March 2020 in India induced a decline in road deaths and homicides, a decrease in unwarranted medical interventions, and a reduction in respiratory ailments from a decrease in pollution following the lockdown are difficult to account for.^38^

## MYTHS UNSUPPORTED BY DATA AND MODELS

### Overstretching Limited Data

Since serosurveys from India indicated that at least 30%–40% of people in large urban areas already have experienced a past infection,^39^ there were many discussions in the scientific community whether India is on its way to reaching herd immunity induced by natural infections. Recent articles^40^ suggest that herd immunity may be impossible to attain and remain an elusive target even with vaccination efforts.

Recent articles suggest that herd immunity may be impossible to attain and remain an elusive target even with vaccination efforts.

Several models had predicted the imminent end of the pandemic at the end of 2020. For example, the government-endorsed supermodel^41^ had predicted that there would not be another surge and that the coronavirus crisis would be substantially over by February 2021. However, intermediate outbreaks in some states refuted this naïve optimism, and the second surge made it clear that there are always possibilities of multiple waves of this virus.^42^

### The Second Wave in India

Since the middle of February 2021, the curve of reported COVID-19 cases in India has risen steeply. Some regions, such as the city of Pune, where serosurveys showed that more than 50% of the population had been infected in the first wave, are currently showing many cases in the second wave.^43^ This sharp rise could be due to a confluence of factors, such as the potential effect of waning immunity (recent studies show 84% protection at 7 months from past infections),^44^ new variants of concern, mass gatherings due to election campaigns, festivities, religious congregations, the reopening of the crowded public transportation system, as well as a sense of false security in the public that has led to a relaxation of preventive measures like face covering and social distancing. While the daily test positivity rate stayed below 2% for much of the period since October 2020, it is currently at 7.2% on March 30, 2021 (covind19.org^5^). Models can data-adaptively capture this oscillatory growth, decay, sharp spikes, and falls by dividing the time series into segments. A new class of models is emerging that aims to not just model the virus transmission but also model changes in human behavior over time through predator-prey models like the Lotka-Volterra model.^45^ We also need to model reinfection, cross-immunity, and the mutation process of the virus to capture the evolution of the epidemic over time.

## SOME PUBLIC HEALTH SUCCESSES FOR INDIA SUPPORTED BY DATA

### Scaling Up Health Care Capacity

Low- and middle-income countries are often denied the same credit for innovation, leadership, and implementation of public health policies as developed nations. Before the pandemic, preparedness indices favored more developed countries, with the UK and the United States listed in the top 5 in the Global Health Security Index (https://www.ghsindex.org). However, even with a modest rank of 57 in the index, India has substantially exceeded expectations, particularly in the way it scaled up testing and treatment facilities during the period of national lockdown in 2020, and managed to reduce overall COVID-19 case-fatality rates from what was expected in 2020.^46^ Partnerships with private laboratories and hospital networks have enabled India to scale up testing from just 3,000 initially to more than 1.8 million tests per day.^47^ The country expanded the ICU bed capacity by 3 times (63,758 in September versus 21,806 in April), the number of isolation beds to 1.55 million in September compared to 173,000 in April, and the number of designated COVID-care centers (15,403 in September versus 1,919 in April).^48^ It is important to stay prepared and continue to build this infrastructure because sweeping surges can happen, as we are noticing with the current oxygen crisis in India's second wave during April 2021.

### Community Engagement Strategies

The public acceptance of masks and nonpharmaceutical interventions in the early months of the pandemic was impressive, given that close social gatherings are an integral part of the cultural fabric of India. The success of community health worker involvement and syndromic surveillance, including in the most affected slum areas of Mumbai,^49^ shows that India's public health approach can provide a unique example for other countries.

The public acceptance of masks and nonpharmaceutical interventions early in the pandemic was impressive, given that close social gatherings are an integral part of the cultural fabric of India.

## BACK TO THE FUTURE: WHAT LIES AHEAD?

The year 2020 ended with at least 3 promising vaccine trials^50^ globally, with multiple vaccine trials going on in India.^51^ Currently, vaccines in development include 45 in Phase I, 33 in Phase II, 23 in Phase III, 6 approved for limited use, and 7 approved for human use. Limited initial supply of vaccines requires countries to adopt model-informed prioritization strategies. Jin et al.^52^ provide a mortality risk score calculator based on various sociodemographic characteristics and predisposing health conditions to prioritize high-risk populations for vaccination in the United States. Bubar et al.^53^ use a mathematical model accounting for vaccine efficacy and age-related variations in susceptibility, immunity, and fatality rates to prioritize available doses. They also consider individual-level serological tests to redirect available doses. Foy et al.^25^ use an age-stratified SEIR-based prediction model to evaluate vaccine allocation strategies in India.

### India's Vaccine Drive

India started one of the largest COVID-19 vaccine drives in the world on January 16, 2021,^54^ within a few weeks of finalizing operational guidelines including prioritization of beneficiaries.^55^ India has approved the Oxford-AstraZeneca vaccine (locally known as Covishield) and the made-in-India vaccine, Covaxin, for emergency use. As of March 30, 61 million doses have been administered, resulting in 0.65% of the population fully vaccinated while 3.8% had received at least one dose.^1^ In March, India administered an average of >2.1 million doses per day (covind19.org^5^). Being one of the largest vaccine manufacturers, India has also donated millions of vaccine doses to neighboring countries as a goodwill gesture and has committed to supplying vaccines to many other countries in the world.^56^ India is expanding the market with emergency use authorizations to other internationally approved vaccines, and vaccines are to be made available to the adult population starting May 1, 2021.

### A COVID-Adaptive Future for India

Emerging new variants of SARS-CoV-2 are predicted to alter the pandemic trajectory around the world in the coming months. For instance, variant B.1.1.7 can bring about another peak in the COVID-19 case counts in the United States despite community vaccination (assuming 1 million vaccine doses are administered per day beginning January 1, 2021, and that 95% immunity is achieved 14 days after 2 doses).^16^ Many other European countries may experience a similar wave of infections from this variant given the expected vaccination rates there.^18^ Recently, a variant with double mutations in the spike protein has been discovered in India, and it is not yet clear if this variant is more or less contagious than the dominant one. However, this is an attractive explanation for the current spike.^57^ A new peak in the COVID-19 trajectory in India is imminent, given the rapidly rising case counts during March 2021 (covind19.org^5^). Strategic genomic sequencing to identify known and emerging variants, accelerating vaccinations with more choices for vaccines (including one shot vaccines), and studying vaccine effectiveness against new variants is going to remain crucial in the coming days.

Follow-up studies of those vaccinated to understand the long-term safety and effectiveness of the vaccines will be necessary. Post-marketing studies for COVID-19 vaccines are all the more important owing to limited premarketing data resulting from their expedited development. Dhanda et al.^58^ highlight the importance of such studies and the key epidemiological considerations, including active surveillance and careful study design. The Indian Council of Medical Research has set up the National Clinical Registry, a cohort of recovered COVID patients.^59^ Monitoring the long-term health of this cohort is crucial as studies have indicated several unexpected post-COVID complications.^60^ Vaccination outcomes in this recovered cohort should be of special interest. The economic recovery process for India will require a much longer time horizon and financial strategy.^61^^,^^62^ Safely reopening educational institutions and providing transitional support and aid to students and teachers will also be key as we look to the future.

## CONCLUSION

The pandemic has underscored structural barriers as well as deep-rooted problems with India's societal and public health infrastructure. It has displayed the inequities and the lack of poor pandemic preparedness in India. It has helped focus our attention on long-standing questions of the quality of public health systems, the need for better data, the importance of communication, and the need for more interdisciplinary expertise to address the so-called "wicked" problems that the current pandemic highlights.^63^ It is imperative to take this as a teaching example and build strong systems to prepare for future pandemics. This requires substantial resource allocations and leadership to strengthen the agenda of health security, especially in the control of communicable diseases. Addressing alarming levels of air pollution,^64^ arresting the high prevalence of noncommunicable diseases, and ensuring adequate support for mental health needs will be pivotal. Investments in public health must increase well above pre-pandemic levels.

Other factors that support a good public health system, including improved health data infrastructure, should be addressed. A planned digital health identity for citizens of India^65^ will help identify elderly individuals, individuals with comorbidities, and essential workers nationwide for vaccination programs.^66^ National-level health record data, together with actionable systems to access and mine this data while maintaining data privacy, will enable a more targeted approach to public health and health care in India. Even in the post-inoculated world, when the case counts reduce to a few hundred, India should have a robust surveillance system to track and contact trace future outbreaks of SARS-CoV-2 infection and identify any new variants. Sustained adoption and incentivization of COVID-appropriate behaviors are going to help us avoid massive lockdowns with crushing economic and social consequences.

## Notes

**Peer Reviewed**

**First published online:** June 7, 2021.
